# The association between attendance of midwives and workload of midwives with the mode of birth: secondary analyses in the German healthcare system

**DOI:** 10.1186/1471-2393-14-300

**Published:** 2014-09-02

**Authors:** Nina Knape, Herbert Mayer, Wilfried Schnepp, Friederike zu Sayn-Wittgenstein

**Affiliations:** Department of Nursing Science, Faculty of Health, University of Witten/Herdecke, Stockumer Str.12, D-58453 Witten, Germany; University of Applied Sciences Osnabrueck, Faculty of Business Management and Social Sciences, Network of Midwifery Research, P.O. 1940, D-49009 Osnabrueck, Germany; University of Applied Sciences Rheine, Frankenburgstraße 31, D-48431 Rheine, Germany

**Keywords:** Midwife, Attendance, Workload, One-to-one, Supportive care, Intrapartum care, Continuity, Caesarean, Mode of birth, Operative delivery

## Abstract

**Background:**

The continuous rise in caesarean rates across most European countries raises multiple concerns. One factor in this development might be the type of care women receive during childbirth. ‘Supportive care during labour’ by midwives could be an important factor for reducing fear, tension and pain and decreasing caesarean rates. The presence and availability of midwives to support a woman in line with her needs are central aspects for ‘supportive care during labour’.

To date, there is no existing research on the influence of effective ‘supportive care’ by German midwives on the mode of birth. This study examines the association between the attendance and workload of midwives with the mode of birth outcomes in a population of low-risk women in a German multicentre sample.

**Methods:**

The data are based on a prospective controlled multicentre trial (n = 1,238) in which the intervention ‘midwife-led care’ was introduced. Four German hospitals participated between 2007 and 2009.

Secondary analyses included a convenience sample of 999 low-risk women from the primary analyses who met the selection criterion ‘low-risk status’. Participation was voluntary. The association between the mode of birth and the key variables ‘attendance of midwives’ and ‘workload of midwives’ was assessed using backward logistic regression models.

**Results:**

The overall rate of spontaneous delivery was 80.7% (n = 763). The ‘attendance of midwives’ and the ‘workload of midwives’ did not exhibit a significant association with the mode of birth. However, women who were not satisfied with the presence of midwives (OR: 2.45, 95% CI 1.54-3.95) or who did not receive supportive procedures by midwives (OR: 3.01, 95% CI 1.50-6.05) were significantly more likely to experience operative delivery or a caesarean. Further explanatory variables include the type of hospital, participation in childbirth preparation class, length of stay from admission to birth, oxytocin usage and parity.

**Conclusion:**

Satisfaction with the presence of and supportive procedures by midwives are associated with the mode of birth. The presence and behaviour of midwives should suit the woman’s expectations and fulfil her needs. For reasons of causality, we would recommend experimental or quasi-experimental research that would exceed the explorative character of this study.

## Background

The continuous increase in caesarean rates across most European countries raises multiple concerns and has led to an on-going debate on the causes of this development. The reasons for these concerns include the increased risks incurred through caesareans, such as repeated operative deliveries for subsequent births and the risk of placenta accreta, placenta praevia or stillbirths in subsequent pregnancies. Germany ranks eighth in the caesarean rate among 28 European countries [1, 2]. Although this development likely depends on a number of factors, including financial reasons, women’s requests and fear of litigation, one possible factor may be the type of care women receive during childbirth.

‘Supportive care during labour’ appears to be an important factor for reducing fear, which should lead to less tension and pain in childbirth [3]. The theoretical framework first described by Grantly Dick-Read describes the elements of fear, tension and pain as a vicious cycle in which every element could be the starting point for this cycle.

Dick-Read explained parts of his theory with the ‘fight-or-flight mechanism’, a prototypic stress response that activates the sympathetic nervous system [4]. In labour, the activation of fear promotes tension of the uterine muscles, and this tension leads to unnecessary pain. Dick-Read described a potential elicitor of the circle of pain, tension and fear: *“It is not infrequently initiated by the two great faults in care of women: loneliness and ignorance.”* [*3:*42]. The provision of ‘supportive care during labour’ seems to be an important factor in reducing stress and the avoidance of pathological states. These conclusions by Dick-Read have been supported by later research [5–7].

More recently, Taylor et al. (2000) have worked on the stress response, showing that effective care by caregivers appears to be related to a female-specific, ‘archaic phenomenon’. Women who feel threatened or fearful become more confident if they are in an established relationship and are affiliated with other social groups, particularly with other women. This phenomenon is a further extension to the popular ‘fight-or-flight mechanism’ and is called ‘tend-and-befriend’ [8].

**Figure 1:**
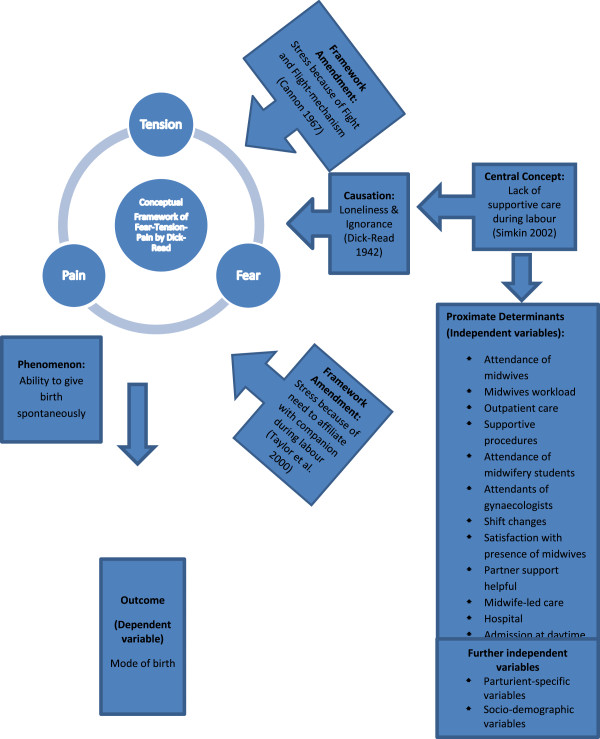
**Theoretical framework.**

Thus, the theoretical framework by Dick-Read and the findings by Taylor underscore the relevance of the concept of ‘supportive care during labour’. A definition of this concept includes the following aspects: giving physical comfort and emotional support to the woman and giving information and instruction as well as advocacy and support for the partner [9]. Central aspects of this concept are similar to the concept by Hunter of ‘being with woman’ [10] or the concept of ‘continuous support’ described by Hodnett et al. [11]. These aspects are also included in ‘the philosophy and model of midwifery care’ by the International Confederation of Midwives [12].The circle of pain, tension and fear by Dick-Read as well as the effects of biobehavioral stress responses and the concept of ‘supportive care during labour’ are illustrated in Figure 1.

‘Supportive care during labour’ requires adequate staffing to implement this concept successfully in maternity units. While international nursing-care studies report strong evidence that workload and time for the patient in hospitals affect quality outcomes [13–18], there is inconsistent evidence with respect to maternity care thus far [11, 19–24].

Several studies have examined the relationship between maternity support and neonatal and maternal outcomes. Many of these studies focus on the concepts of ‘continuity of care’ or ‘one-to-one care’. For example, the Cochrane report by Hodnett et al. related the likelihood of more spontaneous labour with the concept of continuous support. However, differences in cultural contexts, inconsistent starting points of continuous support and differing types of birth attendants in addition to very complex interventions make it difficult to find homogenous results or definitions for these concepts [11, 25].

It is uncertain whether the findings of these studies and the results of the Cochrane review by Hodnett et al., can be applied in Germany because most of them included outcomes for women attended by doulas or other relatively unskilled birth attendants or were conducted in different cultural backgrounds. In the German health care system, it is a legal requirement that every woman is attended by a midwife during childbirth. German midwives are the primary caregiver when contractions start and monitor the progress of labour. Additional companionship by a doula or similar birth attendant is uncommon in Germany.

Supportive care, including the concepts of ‘continuity of care’ or ‘one-to-one-care’ cannot be guaranteed in German hospitals, and women in busy childbirth units might be left alone for long periods of time.

The reasons for a lack of ‘supportive care’ in Germany are, in part, related to healthcare systems within the country. For example, a change in the German Healthcare system introduced a reimbursement concept based on German Diagnosis-Related Groups (G-DRG) in 2003. This regulation forces hospitals to work profitably and efficiently. Personnel costs are an important factor, and there is little doubt that the workload in hospitals has increased over the years [26]. In addition to financial aspects, concentration on more technical and medical aspects in childbirth might have changed the focus and workload of care.

There are no national regulations controlling the maximum workload of midwives. Although a concept of ‘one-to-one care’ is desirable, the concurrent care of many women by one midwife is the daily routine for many German midwives.

Thus, the concept of ‘supportive care during labour’ is closely connected to the healthcare system and healthcare providers. The extent to which ‘supportive care during labour’ can be realised strongly depends on the organisation of the institutions, models of care, staffing (quality and quantity of staff) and overall financial situation. These contextual aspects might influence the outcomes as well. Therefore, the theoretical framework formulated by Dick-Read, and the concept of supportive care during labour must be discussed within the context of the German healthcare system.

This paper reports the association between ‘attendance of midwives’ and ‘workload of midwives’ with the mode of birth outcomes for low-risk women in a German multicentre sample. We examined whether these proximate indicators for the concept of ‘supportive care during labour’ are associated with the mode of birth. In view of the exploratory character of this study, we also considered potential variables that measure other aspects of the concept ‘supportive care’ and covariates that are thought to have an influence on the outcomes.

## Methods

### Study setting, sampling and instruments

The data are based on a prospective controlled multicentre trial including an economic evaluation [27, 28] (n = 1,238). The intervention ‘midwife-led care’ was introduced in the German healthcare system in four different hospitals between 2007 and 2009. The participating hospitals were Reinkenheide/Bremerhaven, Stuttgart/Bad Cannstatt, Asklepios Klinik Barmbek/Hamburg and Asklepios Klinik Harburg/Barmbek. Women were eligible for the study if they had a low-risk status, the fetus was in a cephalic presentation, a vaginal birth was planned and written consent for the study was given. Women who had a caesarean in previous births were eligible if they experienced one spontaneous birth after a caesarean. Participation was voluntary. Ethical approval was obtained from the Medical Chamber Hamburg, Bremen and the Medical Chamber of the federal state of Baden-Württemberg.

For secondary analyses, a convenience sample of 999 women was derived. The considerable share of missing values resulted from the high-quality requirements for data for the variable ‘attendance of midwives’ during labour.

The data were derived from five different documentation tools at three different points of time. A questionnaire on pregnancy (t_1_, n = 1,164) was used to document socio-demographic characteristics. Procedures and outcomes of birth were measured by a self-designed birth documentation tool (a modified document of the official data set of the perinatal registry in Germany) (t_2_, n = 1,238); this tool was complemented by data from hospital-based information systems (t_2_, n = 1,238). In addition, the midwives registered their time with the women and their workload during their shifts using self-constructed time documentation (t_2_, n = 1,225). At eight weeks postpartum, the women received a postal questionnaire (t_3_, n = 1,164 women). For analyses the self-constructed and dichotomous single-item ‘satisfaction with the presence of midwives’ and three scales from the Berne-Basle Childbirth Inventory (BBCI) [Stadlmayr W: *Data Analysis of partner items (BBCI_BEbasic) in 370 women 3-4 days postpartum,* unpublished] were applied from this postal questionnaire to measure satisfaction with support by midwives and partners. The three scales from the BBCI attained a Cronbachs alpha of 0.81 and can therefore be seen as reliable.

All four documentation tools were pretested and checked for face validity. The secondary analysis has adhered to the STROBE guidelines for observational studies.

### Variables for secondary analyses

All of the instruments of the primary analyses identified explanatory variables for the logistic regression model for secondary analyses (Table 1). To prepare the data for the analyses, the dependent variable ‘mode of birth’ was dichotomised. All the spontaneous births defined one group, and all the operative deliveries and unplanned caesareans defined the second group. Due to their low-risk status, only 189 women were part of the operative delivery group, and 810 women experienced spontaneous delivery.

**Table 1:** **The theoretical framework**

	Variables	Instrument
**Proximate indicators of ‘supportive care during labour’**	**Attendance of midwives**	Time documentation tool
	**Workload of midwives**	
	**Outpatient care**	
	**Supportive procedures by midwives**	Birth documentation tool
	**Attendance of a midwifery student**	Time documentation tool
	**Attendance of an obstetrician**	
	**Shift changes**	
	**Satisfaction with the presence of midwives**	Postal questionnaire eight weeks postpartum
	**Partner support helpful**	Postal questionnaire eight weeks postpartum
		(three scales from BBCI)
	**Midwife-led care**	Birth documentation tool
	**Hospital**	Time documentation tool
	**Admission during daytime**	Time documentation tool
	**Birth during daytime**	Time documentation tool
**Parturient-specific variables**	**Parity**	Hospital-based information system
	**Length of stay**	Time documentation tool
	**Epidurals and analgesia**	Birth documentation tool
	**Induction of labour**	
	**Oxytocin usage**	
	**Newborn weight**	Hospital-based information system
	**Childbirth preparation class**	Postal questionnaire postpartum
**Socio-demographic variables**	**Health insurance**	Hospital-based information system
	**Age**	
	**Income**	Willingness-to-pay questionnaire in pregnancy
	**Education**	
	**Partnership**	

The variables ‘attendance of midwives’ and ‘workload of midwives’ were proximate indicators to measure the central aspects of ‘supportive care’. As midwives play a key role in providing professional ‘supportive care during labour’, the increasing workload and lack availability of midwives might be the main reasons for a lack of ‘supportive care’.

The time of attendance was related to the length of stay from admission to birth. A percentage of attendance was measured and dichotomised at the median of 45.6%.

For measuring the workload, we dichotomised whether the capacity of care by a midwife was 100% or otherwise. If midwives were required to care for more than one woman per shift, they were not able to guarantee one-to-one support for a woman.

In addition to these two main variables, other aspects that were also intended to operationalise the concept of ‘supportive care’ or that were thought to influence the concept of ‘supportive care’ directly or indirectly as framing factors were integrated in the analyses.

These factors included the following: outpatient workload per shift, supportive procedures by midwives (massage, aromatherapy (the use of fragrant essential oils), homoeopathy, acupuncture (Chinese medical practice), full bath, kinaesthetic (this is the ability to detect movements of the limbs and body), techniques to optimise positioning or mobilise the woman and partner instruction), attendance of midwifery students, attendance of obstetricians, shift changes, satisfaction with the presence of midwives from the women’s perspective, satisfaction with partner support from the women’s perspective (with a scale for partner support in labour from the Berne-Basel-Inventory-Scale), midwife-led care or consultant-led care, the hospital the woman chose, time of admission and the time of birth (Table 1).

In addition, other parturient and socio-demographic variables were considered in relation to the outcome ‘mode of birth’ (Table 1).

Because of the strict criteria for low-risk pregnancy, no additional variables regarding the prenatal health status were added.

### Statistical analysis

The characteristics of the selected women were described as mean values, the standard deviation, and median values with minimum-maximum intervals for continuous variables and as absolute frequencies and percentages for categorical variables.

For further data analyses, continuous variables were dichotomised at the median or categorised.

To identify variables associated with the ‘mode of birth’, Chi-squared tests were used for categorical variables. All the variables with a p < 0.2 were selected for the final multiple logistic regression model. The high cut-off point of p < 0.2 should help to avoid undetected associations due to confounding factors.

The backward variable selection was used because it lowers the risk of making a type II error [29].

R^2^ values, goodness-of-fit statistics, odds ratios and 95% confidence intervals are reported.

Because only a few values were missing from some participants (5.3% in the multiple logistic regression model), they were not replaced by imputation of substituted values.

The statistical calculations were performed using IBM SPSS statistics, version 20.

## Results

The median and mean age of the study participants was 31 years old (min 18, max 44, SD ±4.64). The average number of children who were already born was 1.41 (min 1, max 6). Nearly all the participants lived in a partnership (90%, n = 899); because of the very small number of women without a partner (n = 19), this variable was not included further. Of all of the women, 4% did not finish school or an apprenticeship. The median monthly income was 1,357 €. Approximately 81% (n = 810) of the sample experienced a spontaneous birth, and 39% (n = 389) received an epidural or analgesia. The median midwifery attendance was 46%. The midwives had a one-to-one workload in only 12% of all cases.

German hospitals also deliver outpatient treatment in the hospital if necessary. In 52% of all cases, midwives delivered outpatient care beyond inpatient care, in the same time period. Of all the births, 47% were accompanied by midwifery students, and 70% of all the women were observed by an obstetrician during childbirth. No shift changes were necessary for 40% of all cases, and 81% of all women were satisfied with the midwives’ presence. Childbirth preparation classes were attended by 77% of the participants, and approximately 54% of the women planned to give birth in a midwife-led care model. The distribution of cases between the four participating hospitals was 15%, 22%, 23% and 40%. The average duration from admission to labour was 385 minutes. Approximately 12% of the women received labour induction, 46% received IV oxytocin and 87% received supportive procedures from midwives, including massage, aromatherapy, homoeopathy, acupuncture, full bath, kinaesthetic and other techniques to optimise positioning or mobilise the patient as well as partner instruction. The descriptive data analyses are presented in Table 2.

**Table 2:** **Description of the study participants in Germany (n = 999)**

Variables		Number	Percent
Mode of birth	Vaginal delivery	810	81.1
	Caesarean/operative delivery	189	18.9
**Proximate indicators of ‘supportive care during labour’**			
Attendance of midwives	≥ 45.60%	499	49.9
	< 45.59%	500	50.1
Workload of midwives	1:1	127	12.7
	Less	872	87.3
Outpatient care	Yes	521	52.1
	No	478	47.8
Supportive procedures	Yes	866	86.7
	No	133	13.3
Attendance of a midwifery student	Yes	474	47.4
	No	525	52.6
Attendance of an obstetrician	Yes	700	70.1
	No	299	29.9
Shift changes	Yes	598	59.9
	No	401	40.1
Satisfaction with the presence of midwives	Yes	809	81.0
	No	137	13.7
	Missing	53	5.3
Partner support helpful	Yes	756	75.7
	No	186	18.6
	Missing	57	5.7
Midwife-led care	Yes	537	53.8
	No	462	46.2
Hospital	A	152	15.2
	B	215	21.5
	C	228	22.9
	D	404	40.4
Admission during daytime	Yes (daytime =7:00 a.m. – 8:59 p.m.)	479	47.9
	No (night-time = 9.00 p.m. – 6.59 a.m.)	520	52.1
Birth during daytime	Yes (daytime =7:00 a.m. – 8:59 p.m.)	583	58.4
	No (night-time = 9.00 p.m. – 6.59 a.m.)	416	41.6
**Parturient-specific variables**			
Parity	Primipara	661	66.2
	Multipara	338	33.8
Time span from admission to labour	≤ 385 min.	501	50.2
	> 386 min.	498	49.8
Usage of epidurals and/or analgesia	Yes	389	38.9
	No	610	61.1
Labour induction	Yes	123	12.3
	No	876	87.7
Oxytocin	Yes	460	46.0
	No	539	54.0
Newborn weight	≤ 3,470 g	418	41.8
	> 3,471 g	411	41.1
	Missing	170	17.0
Childbirth preparation class	Yes	771	77.2
	No	175	17.5
	Missing	53	5.3
**Socio-demographic variables**			
Health insurance	Public	869	87.0
	Private	49	4.9
	Missing	81	8.1
Age	18-29	409	40.9
	30-34	378	37.8
	35-39	166	16.6
	≥ 40	46	4.6
Income	≤ 1,356.52 €	366	36.6
	> 1,356.53 €	522	52.3
	Missing	111	11.1
Educational level	School without apprenticeship	44	4.4
	Apprenticeship	507	50.8
	Academic	363	36.3
	Missing	85	8.5
Family status	With partner	899	90.0%
	Without partner	19	1.9%
	Missing	81	8.1%
Total		999	100.0%

Bivariate analyses of a set of 25 variables revealed that the mode of birth was not significantly influenced (p > 0.2) by the outpatient workload, satisfaction with partner support, admission or birth during daytime, private health insurance or newborn weight; these factors were excluded for the multivariate model.

The remaining 19 variables were significantly (p < 0.2) associated with the ‘mode of birth’, including ‘attendance of midwives’ and ‘workload of midwives’ (Table 3).

**Table 3:** **Bivariate analyses (Chi-squared tests) for potential explanatory variables (n = 999)**

		Caesarean or operative delivery		Percent of deliveries with caesarean or operative delivery	Chi-squared test (significance p < 0.2)
		No	Yes		
**Proximate indicators of ‘supportive care during labour’**					
Attendance of midwives	≥ 45.60%	433	66	13.2	<.001***
	< 45.59%	377	123	24.6	
Workload of midwives	1:1	113	14	11.0	.010*
	Less than 1:1	697	175	20.1	
Outpatient care	Yes	425	96	18.4	.678
	No	385	93	19.5	
Supportive procedures	Yes	696	170	19.6	.131
	No	114	19	14.3	
Attendance of a midwifery student	Yes	355	119	25.1	<.001***
	No	455	70	13.3	
Attendance of an obstetrician	Yes	511	189	27.0	<.001***
	No	299	0	0.0	
Shift changes	Yes	440	158	26.4	<.001***
	No	370	31	7.7	
Satisfaction with the presence of midwives	Yes	679	130	16.1	<.001***
	No	84	53	38.7	
Partner support helpful	Yes	604	152	20.1	.281
	No	155	31	16.7	
Midwife-led care	Yes	452	85	15.8	.007**
	No	358	104	22.5	
Hospital	A	134	18	11.8	<.001***
	B	187	28	13.0	
	C	190	38	16.7	
	D	299	105	26.0	
Admission during daytime	Yes	392	87	18.2	.558
	No	418	102	19.6	
Birth during daytime	Yes	472	111	19.0	.908
	No	338	78	18.8	
**Parturient-specific variables**					
Parity	Primipara	481	180	27.2	<.001***
	Multipara	329	9	2.7	
Time span from admission to labour	≤ 385 min.	472	29	5.8	<.001***
	> 386 min.	338	160	32.1	
Epidural/opiates	Yes	248	141	36.2	<.001***
	No	562	48	7.9	
Induction of labour	Yes	88	35	28.5	.006**
	No	722	154	17.6	
Oxytocin	Yes	309	151	32.8	<.001***
	No	501	38	7.1	
Newborn weight	≤ 3,470 g	329	89	21.3	.406
	> 3,471 g	333	78	19.0	
Childbirth preparation class	Yes	597	174	22.6	<.001***
	No	166	9	5.1	
**Socio-demographic variables**					
Health insurance	Public	711	158	18.2	.285
	Private	37	12	24.5	
Age	18-29	344	65	15.9	.131
	30-34	293	85	22.5	
	35-39	135	31	18.7	
	≥ 40	38	8	17.4	
Income	≤ 1,356.52 €	330	36	9.8	<.001***
	> 1,356.53 €	389	133	25.5	
Educational Level	School without apprenticeship	42	2	4.5	.002**
	Apprenticeship	419	88	17.4	
	Academic	279	84	23.1	

*p < .05, **p < .01, ***p < .001.

The multivariate regression analysis revealed statistically significant associations with ‘mode of birth’ for eight variables (Table 4). Women receiving no supportive procedures from midwives were more than three times more likely to have an operative delivery or caesarean (Odds Ratio (OR): 3.01, 95% CI 1.50-6.05), and women dissatisfied with the presence of the midwives were more than 2.5 times more likely to have an operative delivery or caesarean (OR: 2.45, 95% CI 1.53-3.93).

**Table 4:** **Results of multivariate analyses for the outcome mode of birth using backward logistic regression (n = 946)**

			95% CI for Odds Ratio		
		B (SE)	Lower	Odds Ratio	Upper
Included					
Constant		-5.73 (0.57)		0.00	
**Proximate indicators of ‘supportive care during labour’**					
Supportive procedures by midwives	Yes			1.0	
	No	1.10 (0.36)	1.50	3.01**	6.05
Satisfaction with the presence of midwives	Yes			1.0	
	No	0.90 (0.24)	1.53	2.45**	3.93
Hospital	A (Reference)			1.0	
	B	0.33 (0.37)	0.68	1.39	2.86
	C	0.03 (0.35)	0.52	1.03	2.05
	D	0.99 (0.32)	1.45	2.70**	5.02
**Parturient-specific variables**					
Parity	Primipara	1.44 (0.39)	1.95	4.20***	9.07
	Multipara			1.0	
Time span from admission to labour	≤ 385 min.			1.0	
	> 386 min.	1.00 (0.258)	1.63	2.71***	4.49
Epidural/opiates	Yes	0.86 (0.23)	1.51	2.35***	3.66
	No			1.0	
Oxytocin	Yes	0.92 (0.24)	1.57	2.51***	4.01
	No			1.0	
Childbirth preparation class	Yes	0.71 (0.40)	0.93	2.04	4.47
	No			1.0	

R^2^ (Homer & Lemeshow) = .52, Nagelkerkes R^2^ = .37, Cox & Snell .23, χ^2^ = 249.54, **p < .01, ***p < .001.

Compared to reference hospital A, only women in hospital D were significantly more likely to experience an operative birth or a caesarean (OR: 2.70, 95% CI 1.45-5.02).

Women with an epidural or opiate were more than twice as likely (OR: 2.35, 95% CI 1.51-3.66) and women whose labour was augmented with oxytocin were 2.5 times more likely to have a caesarean or operative delivery (OR: 2.51, 95% CI 1.57-4.01).

Women who had longer time spans from admission to labour (OR: 2.71, 95% CI 1.63-4.49) and women who took part in childbirth preparation class were at increased risk for caesareans and operative deliveries (OR: 2.04, 95% CI 0.93-4.47).

The strongest association was with parity. Nulliparous women were four times more likely to have a caesarean or an operative delivery compared with multipara women (OR: 4.20, 95% CI 1.95 – 9.07).

Midwives’ attendance and workload did not contribute to the model.

The variable ‘presence of an obstetrician’ was excluded from the logistic regression model due to the statistical effect of complete separation [30].

Both income and educational level were excluded from the first model due to significant missing data. An additional model including income and educational level revealed similar results (data not shown).

## Discussion

In line with previous international studies that evaluated the effects of intrapartum nurses and midwives on the mode of birth, no association was found between ‘attendance of caregivers’ or ‘workload’ and the mode of birth [23, 24, 30]. Nevertheless, the work of midwives in general was associated with the mode of birth, and significant effects were identified for the self-rated satisfaction of women with the presence of midwives.

Women who were not satisfied were more than twice as likely to have a caesarean or operative delivery. These data reflect the possible effect of the presence of midwives on the mode of birth and might be a more efficient proxy indicator for the interaction between women and midwives.

The lack of any ‘supportive procedures by midwives’, including massage, aromatherapy, homoeopathy, acupuncture, full bath, administration of an enema, kinaesthetic, other techniques to optimise positioning or mobilise the woman and partner instruction, was associated with a threefold increase in the incidence of a caesarean or operative delivery.

Professional ‘supportive care during labour’ appears to be an important factor for effective care. Moreover, findings suggest that different care philosophies and care standards in hospitals might lead to varying probabilities for the mode of birth and might mirror different strategies of ‘supportive care’.

Our observations regarding parity and its negative relationship with caesarean or operative delivery has been described before [31–33]. The present findings may have been influenced by the selection criteria because only multipara women who had already experienced spontaneous birth were included in the sample. Thus, the likelihood of having a spontaneous labour was very high, and only nine multipara women experienced a caesarean.

Consistent with previous research, the use of epidurals and opiates and the time span between admission and birth were positively related to the likelihood of caesarean and operative delivery [23, 32, 34–38].

Although the literature are inconsistent with respect to the usage of oxytocin and its effect on the mode of birth [32, 39–41], we observed a negative relationship between the likelihood of spontaneous birth and the usage of this hormone. Oxytocin usage in Germany is common if dystocia or bradytocia occurs and is used to a lesser degree for active management during labour. The administration of oxytocin in our study might be a proxy indicator for an abnormal birthing progress, whereas its administration in other controlled trials may have been an aspect of active labour management.

Furthermore, inconsistent results were observed in the literature with respect to the effectiveness of participation in childbirth preparation class [42–46]. In our regression model, participation slightly but not significantly contributed to the likelihood of caesarean or operative delivery. An explanatory factor might be that nulliparous and anxious women are more likely to take part in preparation classes. Nulliparous and anxious women might also be at higher risk of experiencing fear, tension and pain and therefore might be of higher risk of a caesarean.

Socio-demographic covariates, such as age, income and educational level, were not associated with the mode of birth. This result is consistent with other German studies [41, 47] but is inconsistent with international studies [23, 32, 33, 38, 48–50].

### Limitations

To permit comparability, the sample was restricted to low-risk women. In a prospective study, a sample should also include high-risk women and reflect their needs.

The use of ‘mode of delivery’ as an optimal endpoint to measure the effects of ‘supportive care during labour’ is questionable. Whether midwives change their supportive behaviour or attendance in the birthing process if progress in labour begins to accelerate or decelerate has not been established. If women do not obtain significant support at the beginning and pathological progression occurs, midwives might change their attitude toward the woman and give her more support/more attendance. However, women who cope well with labour will not require as much support as women who experience significant pain, tension and perhaps dystocia. Thus, attendance during labour might depend on the situation and on the birthing process. The dynamic process of labour is determined by many factors and has been reported in another context by Groß [51].

Another limitation might be the fact that attendance was measured from admission to delivery, and this time span could be insufficient to explain effects on the mode of birth. For a prospective study, a consistent starting point (e.g., established labour) is recommended.

The variable ‘workload’ was restricted to the information regarding whether midwives were responsible for more than one woman in their shift. The variable did not reflect whether this workload was parallel to the study case, and the time span for this additional case was not restricted. A prospective measurement of workload should be realised with more precise instruments.

Although ‘satisfaction with midwives’ presence’ was significantly associated with the mode of birth, it cannot be precluded that birth experience influenced the women’s evaluation.

To gather more non-ambiguous results, a primary analysis with an experimental design is preferred. Because of the given non-causality of secondary analyses and the restriction in variables, a primary analysis might overcome these restrictions. However, ethical issues must be clarified for a randomised controlled trial. Indeed, how to randomise women such that one group receives less ‘supportive care’ must be discussed.

Nonetheless, the convenience sample was consistent with data from the German perinatal registry. The prevalence of spontaneous births was 81.1% in this low-risk sample, which is consistent with national data from 2010 if unplanned caesareans, children with a birth weight > 1,500 g and cephalic presence are considered (78.9%) [52]. However, the sample was not representative of Germany with regard to all aspects: although approximately 20% of mothers in the German population are highly educated [53], our sample included more than 36.3% highly educated mothers.

## Conclusions

To our knowledge, this is the first German study exploring how ‘supportive care during labour’ by midwives is associated with the mode of birth. The hypothesis that the parameters ‘attendance’ and ‘workload’ influence the outcome directly must be rejected. Nevertheless, the study was able to document that other aspects of ‘supportive care during labour’ are associated with the mode of birth. Summing up: professional ‘supportive care’ by midwives appears to be helpful, preventing caesarean and operative delivery. The reasoning by Dick-Read that loneliness and ignorance in maternity care are one of the main causes for increasing pain, tension and fear can be supported by these results. Thus, the circle of fear, tension and pain hypothesised by Dick-Read might be interjected or reduced if aspects of supportive care increase.

However, women’s perceptions of the attendance of midwives are more meaningful than the objective measurements of attendance or workload. The presence and behaviour of the midwife must suit the woman’s needs and fulfil her demands. A further aspect of the work of midwives is the application of supportive techniques (massage, aromatherapy, homoeopathy, acupuncture, full bath, administration of an enema, kinaesthetic and other techniques to optimise positioning or to mobilise women and partner instruction). This support is associated with a lower incidence of caesarean or operative deliveries and might mirror the effect of midwives’ professional skills.

In conclusion, ‘supportive care in labour’ represents a very complex concept. The operationalisation of such a concept is limited by the availability of data in the dataset. Ross-Davie et al. have also suggested that evaluating ‘professional intrapartum support’ requires methods that reliably measure the quantity and quality of this concept [54].

For reasons of causality and complexity, we would recommend more experimental or quasi-experimental research that exceeds the explorative character of this study.

## Abbreviations

BBCI: Berne-Basel-Inventory-Scale
G-DRG: German Diagnosis Related Groups
OR: Odds ratio.
